# Activation of intervertebral disc cells by co-culture with notochordal cells, conditioned medium and hypoxia

**DOI:** 10.1186/1471-2474-15-422

**Published:** 2014-12-11

**Authors:** Benjamin Gantenbein, Elena Calandriello, Karin Wuertz-Kozak, Lorin M Benneker, Marius JB Keel, Samantha CW Chan

**Affiliations:** Tissue & Organ Mechanobiology, Institute for Surgical Technology and Biomechanics, University of Bern, Bern, Switzerland; Competence Center for Applied Biotechnology and Molecular Medicine, University of Zurich, Zurich, Switzerland; AOSpine Research Network, Duebendorf, Zurich Switzerland; Institute for Biomechanics, ETH Zurich, Zurich, Switzerland; Department for Orthopaedic Surgery, Inselspital, University of Bern, Bern, Switzerland; Bioactive materials, EMPA, Swiss Federal Laboratories for Materials Science and Technology, St Gallen, Switzerland

**Keywords:** Co-culture, Conditioned-medium, Notochord, Nucleus pulposus, Proteoglycan/DNA content, Relative gene expression, Mass spectrometry

## Abstract

**Background:**

Notochordal cells (NC) remain in the focus of research for regenerative therapy for the degenerated intervertebral disc (IVD) due to their progenitor status. Recent findings suggested their regenerative action on more mature disc cells, presumably by the secretion of specific factors, which has been described as notochordal cell conditioned medium (NCCM). The aim of this study was to determine NC culture conditions (2D/3D, fetal calf serum, oxygen level) that lead to significant IVD cell activation in an indirect co-culture system under normoxia and hypoxia (2% oxygen).

**Methods:**

Porcine NC was kept in 2D monolayer and in 3D alginate bead culture to identify a suitable culture system for these cells. To test stimulating effects of NC, co-cultures of NC and bovine derived coccygeal IVD cells were conducted in a 1:1 ratio with no direct cell contact between NC and bovine nucleus pulposus cell (NPC) or annulus fibrosus cells (AFC) in 3D alginate beads under normoxia and hypoxia (2%) for 7 and 14 days. As a positive control, NPC and AFC were stimulated with NC-derived conditioned medium (NCCM). Cell activity, glycosaminoglycan (GAG) content, DNA content and relative gene expression was measured. Mass spectrometry analysis of the NCCM was conducted.

**Results:**

We provide evidence by flow cytometry that monolayer culture is not favorable for NC culture with respect to maintaining NC phenotype. In 3D alginate culture, NC activated NPC either in indirect co-culture or by addition of NCCM as indicated by the gene expression ratio of aggrecan/collagen type 2. This effect was strongest with 10% fetal calf serum and under hypoxia. Conversely, AFC seemed unresponsive to co-culture with pNC or to the NCCM. Further, the results showed that hypoxia led to decelerated metabolic activity, but did not lead to a significant change in the GAG/DNA ratio. Mass spectrometry identified connective tissue growth factor (CTGF, syn. CCN2) in the NCCM.

**Conclusions:**

Our results confirm the requirement to culture NC in 3D to best maintain their phenotype, preferentially in hypoxia and with the supplementation of FCS in the culture media. Despite these advancements, the ideal culture condition remains to be identified.

**Electronic supplementary material:**

The online version of this article (doi:10.1186/1471-2474-15-422) contains supplementary material, which is available to authorized users.

## Background

**Figure 1:**
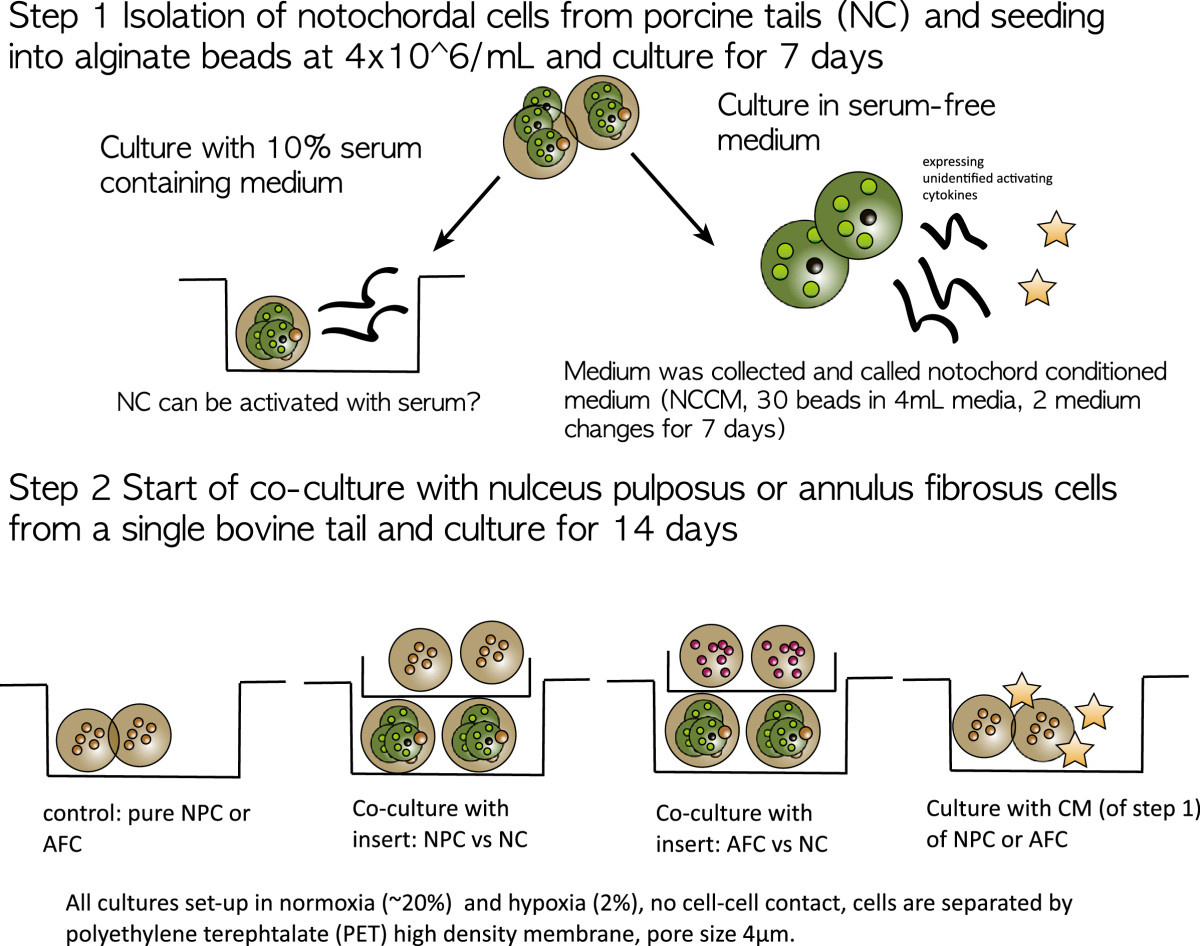
**Experimental set-up to test NC regenerative effects on co-culture with NPC or AF that were stimulated initially in pre-culture with and without 10% fetal calf serum (FCS) and subsequent co-culture or exposure with conditioned medium (NCCM) from step 1.** The design also involved a comparison of normoxia (~20% oxygen) and hypoxic conditions (2% oxygen).

The notochord is a key structure in early embryogenesis in all chordates and is populated with relatively big and vacuole enriched notochordal cells (NC)[1, 2]. The cells form the center of the intervertebral disc (IVD) in the fetus and the adult[3, 4]. These cells are either present throughout the lifetime at various ratios across the vertebrate phylogenetic tree with respect to other IVD cells, or they are reduced to a minimum population after adolescence[5, 6]. In humans, it has been found that they disappear early in childhood[7]. It has been described that in different mammalian groups, these cells co-exist with nucleus pulposus cells (NPC) at different ratios[5, 7]. For instance, in cattle, goats, and sheep, these cells disappear early in lifetime[3, 7], whereas in rodents (rats and mice) and rabbits, a high number of NC is maintained throughout their lifetime. In dog breeds, both IVD types are present. In the so-called non-chondrodystrophic dogs (e.g. larger dogs, such as greyhounds) in which low back pain is less common, a high ratio of NC is present. Conversely, in chondrodystrophic dog breeds (e.g. beagle, dachshund), few NC are present, and these breeds frequently suffer from disc degeneration and low back pain. Over the past decade, the fate of NC has been a matter of debate. It was hypothesized that these cells either disappear by apoptosis or that they are differentiated into an IVD chondrocyte-like cell phenotype[8, 9]. On this matter, recent transcriptome analyses of the possible origin of these cells have pointed into the direction of precursor origin and that these two cell populations — although very different in size and granularity — were not so different in their phenotypic profile[10, 11]. The open question remains about the mesodermal or ectodermal origin of these cells[12–14]. However, recently, “noto,” a highly conserved homeobox gene restricted to the notochord, where it regulates node morphogenesis, has been tracked by a novel notochord-specific Cre mouse. These gene-tracking data, during embryogenesis, clearly demonstrated that the notochord indeed gives rise to the NP and later, to the smaller chondrocyte-like disc cells, which confirms the close relationship of the NC and the smaller NPC[15–17]. However, the interaction between these morphologically different cell types remains obscure, hence leading to cross-species co-cultures investigations[13, 14, 18] and conditioned medium experiments[13, 14, 19–23]. In a dog model, it was found that NC secrete connective tissue growth factor (CTGF, syn. CCN2) and can protect NPC from undergoing apoptosis[24, 25] by inhibiting the caspase pathway. It was furthermore demonstrated that NC are highly sensitive to the culture conditions, i.e., to media composition and oxygen content[26]. More importantly, NC or their cultured media — the so-called notochord-derived conditioned medium (NCCM) — demonstrated regenerative effects by inducing the glycosaminoglycan per DNA (GAG/DNA) ratio[13, 18, 21]. These cells, or, possibly, merely their released factors, are of high interest for regenerative medicine to re-populate degenerated IVDs or to stimulate native disc cells. Our aim was to test whether NC can activate NPC in vitro and which initial culture conditions may be favorable to activate more differentiated disc cells. The experimental set-up (explained below) is shown in Figure 1. Our first step was to revisit the question for suitable culture conditions for these cells. Previous cell culture studies expanded NC in 2D monolayer[14] and also, more recently, in 3D[26, 27]. It has been noted that NC are highly sensitive to mechanical loading and changes to the nutritional environment[26–29]. What is currently missing is a direct proof that 2D culture conditions are indeed unfavorable for NC and that the activation capacity of NC on other cells is dependent on the nutritional state of the NC in culture. Thus, we wanted to demonstrate the effect of 2D monolayer culture versus 3D cell culture in alginate. Furthermore, we investigated the hypothesis that NC are sensitive to culture conditions[28] and that their activation capacity on disc cells in indirect co-culture experiments can be stimulated by exposure to fetal calf serum (FCS). As a positive control, we included the previously described application of conditioned medium (NCCM) collected from NC that has been demonstrated to stimulate smaller NPC[21, 22, 27, 30]. However, it is unknown whether annulus fibrous cells (AFC) can be activated by either co-culture or exposure to NCCM. We then also replicated the experiments in normoxic (~21%) and hypoxic (~2%) conditions to study the effect of the physiological oxygen environment as it was also reported in the literature that NC should be preferentially cultured in hypoxia for a better aligned extracellular matrix[20, 26] and a more authentic phenotype as defined by the production of aggrecan with respect to collagen type 2, which was found to be around 27:1 in human tissue[31] or around 1:1 as found in rabbit tissue[32].

## Methods

### Cell source and expansion

The post-mortem usage of biological material for biomedical research taken from the food chain requires no ethical permit according to the Swiss and European law. Porcine notochordal cells (NC) were isolated from the nucleus pulposus (NP) tissue of porcine tails obtained from 4- to 5-month-old pigs collected from the local abattoir. Tails with a high percentage of NC (~80%) were selected for the experiment. The percentage of NCs was assessed and counted using a haemocytometer and brightfield microscopy to confirmed the NC phenotype as large in cell size and presence of vacuoles. Bovine nucleus pulposus cells (NPC) were harvested from the NP tissue of ~1-year-old bovine tails obtained from the local slaughter house. Porcine and bovine NP tissues were digested by 0.19% pronase (Roche, Basel, Switzerland) for 1 hour and subsequent collagenase type 2 at a concentration of 32 U/mL in 25 mL of HG-DMEM supplemented with 10% FCS (Worthington, London, UK) overnight (~14 hours) at 37°C and standard culture conditions (5% CO2 and 100% humidity)[14, 23].

### Cell separation using micro sieves and cytometry using FACS

**Figure 2:**
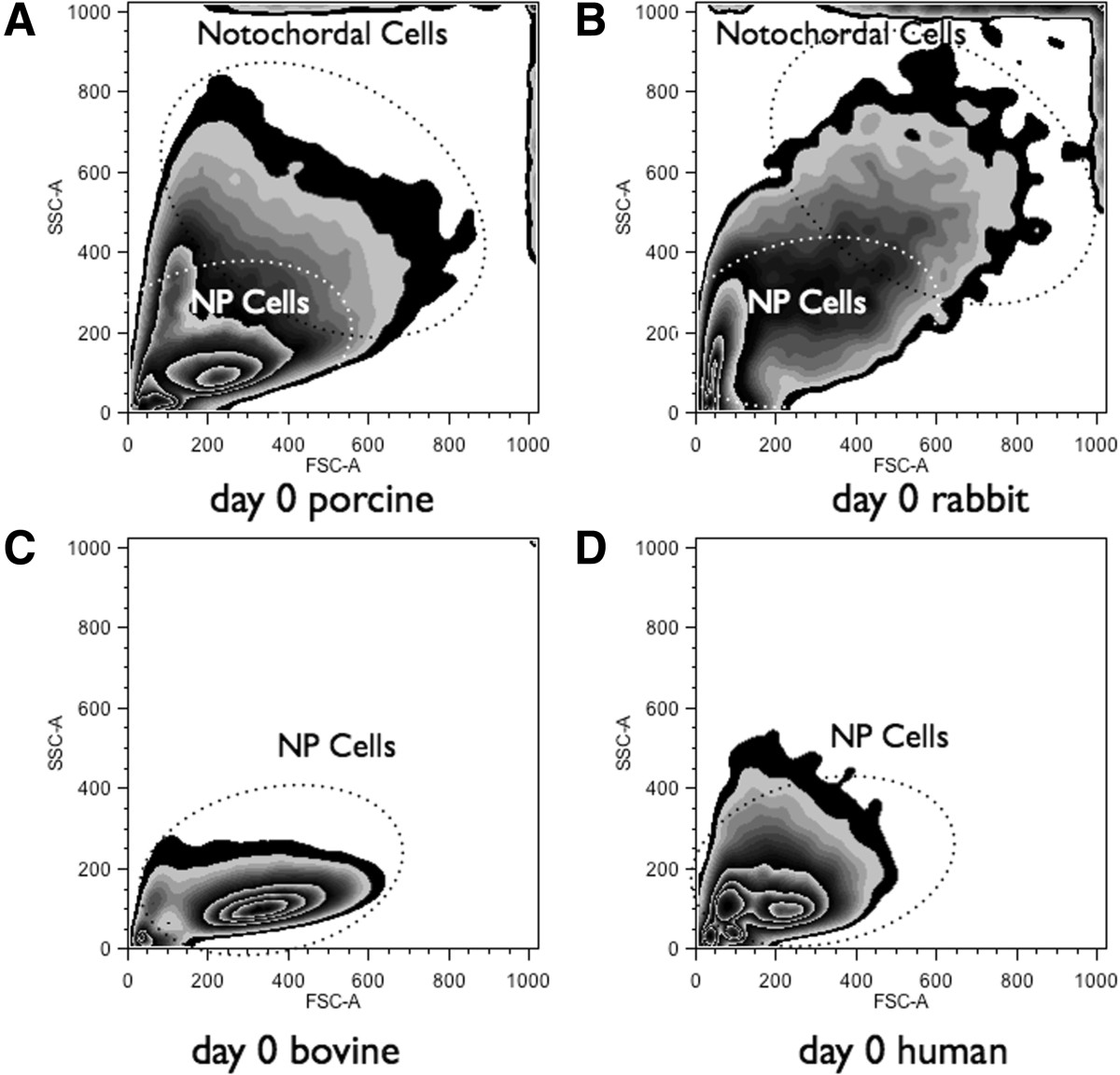
**Flow cytometry of unlabeled primary cells isolated from intervertebral discs (IVD) of four different species. A**. Porcine passage 0 coccygeal IVD cells **B**. Rabbit passage 0 lumbar IVD cells. **C**. Bovine passage 0 coccygeal IVD cells, and **D**. Human passage 0 lumbar IVD cells (L1-2). Note the presence of notochordal cells, which are bigger and spread higher on both forward scatter and side scatter in **A**. and **B**. Parameters of flow cytometer were constant for all measurements. Axes are FSC-A = forward scatter, SSC-A = side scatter.

Cells isolated from porcine NP tissues were separated with three cell sieves, i.e., cells > 20 μm, 8-20 μm and cells < 8 μm (CellMicroSieves, BioDesign Inc., New York, USA)[33] with the goal to identify NPC and NC. To characterize and confirm the cell populations by size, the filtered and unlabeled cell fractions were resuspended in phosphate buffered saline (PBS) containing 0.5% bovine serum albumin (BSA) and were then analyzed on a fluorescence-activated cell sorting (FACS) device (LSR II H271, Bdbiosciences, inc., Allschwil, Switzerland) with 429V for forward scattering (FSC-A) and 308V for side scattering (SSC-A). Gates were analyzed and visualized using FlowJo software 10.07R2 (TreeStar, inc., Ashland, OR).Primary gates (Figure 2) for small pNPC and bigger notochordal cells (pNC) were defined based on primary cells digested from discs lacking a notochordal cell phenotype, including bovine and a whole human lumbar IVD obtained from a spinal fusion (ethical permit has been obtained from the local ethical committee). The size of these cells was compared to the cell size of NC isolated from rabbit IVD and porcine IVD. Cells were grown in 2D monolayers for three passages or in 3D alginate beads at a density of 4M/mL alginate in Dulbecco’s High Glucose Modified Eagle Medium (HG-DMEM, containing 4.5 g/mL glucose, Gibco, Lucerne, Switzerland) +10% FCS in order to test for cell size shifts during the cell expansion and to compare 2D monolayers with 3D alginate cultures.

### 3D cell encapsulation and co-culture

The cells were re-suspended in 1.2% alginate at a density of 4 M/mL and embedded in ~30 μl beads by pressing the suspension at a constant rate through a syringe (22G needle) and dropping them into a 102 mM CaCl_2_ salt solution[34]. The porcine NC:NPC ratio in the tissue was estimated to be ~80% notochordal[23]. The cells were kept in co-cultures of NC: NPC ratios of 1:1 in serum-free HG-DMEM medium containing 100 μg/mL penicillin/streptomycin, 50 μg/mL ascorbic acid, ITS+ and non-essential amino acids or in HG-DMEM supplemented with 10% fetal calf serum (all from Sigma-Aldrich, Buchs, Switzerland) (Figure 1). All bead-bead co-cultures were conducted in duplicate in 12-well plates, using 0.4 μm pore size, high pore density, polyethylene terephthalate (PET) track-etched culture inserts (Becton, Dickinson and Company, Allschwil, Switzerland). The co-cultures were tracked on day 0, day 7, and day 14. Day 0 was defined as the day of cell embedding into the 3D alginate environment. There were four co-culture pairings (thus, each N = 4 for the porcine and bovine animals) (see also Figure 1). The cultures were either kept in normoxia (20% atmospheric oxygen) or in hypoxia using a C-274-2-shelf chamber inside a standard incubator and 1x Pro-Ox controller, (Biospherix, inc., NY) and keeping O_2_ at 2% by addition of N_2_.

### Metabolic activity

Cell activity of the cells in alginate beads was measured using resazurin assay[35]. Two beads per condition were incubated in 500 μL DMEM with 10% FCS and 50 μM resazurin sodium salt (Sigma-Aldrich, Buchs Switzerland) for 3 hours in a 48-well plate[23]. The relative fluorescence unit (RFU) was then measured at an excitation wavelength of 547 nm and an emission wavelength of 582 nm using a Softmax pro multi-wave length fluorescence reader (Molecular Devices, distributed by Bucher Biotec, Switzerland). RFU values were expressed relative to the activity measured on day 0, i.e. the day of seeding the cells into alginate beads.

### Quantification of GAG and DNA content

Alginate beads from the resazurin assay were digested with papain (Sigma-Aldrich, Buchs, Switzerland) overnight at 60°C. The samples digested with papain were used for glycosaminoglycan (GAG) and DNA measurement. The GAG content was measured by the modified dimethylmethylene blue (DMMB) assay (pH 1.5)[36, 37]. The absorbance of the samples added to the DMMB buffer was read at 595 nm with a fluorescence reader (Molecular Devices). GAG concentrations were calculated from a standard curve obtained with chondroitin sulfate (Sigma-Aldrich, Buchs, Switzerland). The amount of DNA in the sample was measured with bisbenzimidol fluorescent dye (Hoechst 33258, Sigma-Aldrich). Fluorescence was again detected with a multi-wavelength fluorescence reader (Molecular Devices). A standard curve was generated with known concentrations of calf thymus DNA (Sigma-Aldrich, Buchs, Switzerland), and the amount of DNA of each sample was calculated from the standard curve.

### Real-time RT-PCR

Relative gene expression of major anabolic genes of to the extracellular matrix turnover was monitored, i.e., aggrecan (ACAN), collagen type 1 and 2 (Col 1 and Col 2, respectively) and ribosomal 18S used as a reference gene[38–40]. Typical nucleus pulposus and notochordal cell markers including brachyury (T) and CD24[41] were monitored. Due to DNA substitutions between the porcine and bovine codon sequences of one of the two primer regions, species-specific RT-PCR were designed (Table 1) for both NCs and NPCs. Relative gene expression was estimated by application of a threshold cycle (Ct) and calculation of ∆∆Ct and the statistics of the 2-∆∆Ct according to Livak & Schmittgen[42].

**Table 1:** **Primer sequences used for relative real-time RT-PCR**

Gene	Accession number	Forward (5′-3′)	Reverse (5′-3′)
bovine *(Bos taurus)*			
Bt_r18S	DQ222453.1	ACG GAC AGG ATT GAC AGA TTG	CCA GAG TCT CGT TCG TTA TCG
Bt_ACAN	NM_173981.2	GGC ATC GTG TTC CAT TAC AG	ACT CGT CCT TGT CTC CAT AG
Bt_CD24	NC_007300.4	AGA CTT ACT CAA ATC AAA	AAC AGT AGA GAT GTA GAA
Bt_col1 A2	NM_174520.2	GCC TCG CTC ACC AAC TTC	AGT AAC CAC TGC TCC ATT CTG
Bt_col2 A1*	AY743675.1	CGG GTG AAC GTG GAG AGA CA	GTC CAG GGT TGC CAT TGG AG
porcine *(Sus scrofa domestica)*			
Ssd_r18S	NR_046261.1	TAG AAG GAA GAG GAA CCA T	TAA TGT CCA ACT CAC TGA AG
Ssd_ACAN	NM_001164652.1	CAG TAA CTT CGT GCC TAG	GGT CCT CTA TCT CCA GTT
Ssd_T (brachyury)*	NC_010443.4	TCA CCA ACA AG CTCA ATG	CTC TCA CGA TGT GGA TTC
Ssd_col1 A2	AB237775.1	TAT CGG AAT TAA CCA GAC A	ACA GGA TTG ACA GAT TGA

*Denotes primer match for both species.

The PCR was run in a two-step protocol; 45 cycles with a 2-step protocol and an annealing temperature at 57°C.

### Mass spectrometry analysis and protein identification of conditioned medium

Proteins of 1) serum-free control medium versus 2) NCCM and 3) medium collected from NPC and NC co-culture were lysed in SDS-PAGE sample buffer and proteins separated on 10% SDS-PAGE gels. Gels were stained with Sypro Ruby® protein gel stain (Sigma), and the separated proteins were isolated from the gel according to Heller et al.[43]. Briefly, peptide sequencing was made on a LTQ Orbitrap XL mass spectrometer (ThermoFisher Scientific, Bremen, Germany) equipped with a Rheos Allegro nano flow system with AFM flow splitting (Flux Instruments, Reinach, Switzerland) and a nano electrospray ion source operated at a voltage of 1.7 kV. Peptide separation was performed on a Magic C18 column (5 μm, 100 Å, 0.075 × 70 mm) using a flow rate of ~400 nL/min and a linear gradient of 5 to 40% acetonitrile in water/0.1% (v/v) formic acid over 60 min. Data acquisition was also done according to Heller et al.[43]. Using the Hardklör Database, mascot generic files were generated[44]. Applying a two round strategy, the searches were done with Phenyx[45] against the forward and reversed UniprotKB SwissProt protein database (Release 2013_12) of human entries. The parameters for the peptide search on the SwissProt protein database were also conducted according to Al Kaabi et al.[46]. The peptide match score summation (PMSS) value for each identified protein was used as a semi-quantitative abundance estimate. PMSS values were normalized by division with the median PMSS across all proteins in each sample and multiplication with the median across all samples. We use the SwissProt ID as the protein name in this manuscript.

### Histology

The alginate beads were initially fixed in Tissue-Tek® O.C.T.™ compound (Sakura Finetek Europe, Leiden, The Netherlands) for 20 min and then snap-frozen in liquid nitrogen. Beads were then sectioned in 12 μm slices with a cryotome (Microm HM560, Thermoscientific, Reinach, Switzerland). For immune-staining, slices were incubated in solutions containing 0.1M CaCl_2_ to prevent dissolution of beads. The cells were first permeabilized with 100% methanol for 2 min and then blocked with 10% FBS 0.1M CaCl_2_ for 1 hour. The beads were then incubated with polyclonal rabbit brachyury (H-210) polyclonal primary antibody for 1h at a 1:200 dilution (sc-20109, Santa Cruz biotech, Santa Cruz, CA, USA). After washing, the primary antibody was visualized with the secondary antibody Alexa Fluor® 488 Goat anti-Rabbit IgG antibody (A1108, Molecular Probes, Invitrogen, Basel, Switzerland) at a 1:500 dilution. Sections were mounted in ProLong® Gold Antifade Reagent with DAPI (P-36931, Molecular Probes) to stain the nuclei and were visualized on an inverted fluorescent microscope (Nikon Eclipse E800, Nikon Instruments, Tokyo, Japan).

### Statistical analyses

All data are given as relative to the “pure” cell population of the same culture day. “Pure” is defined as primary isolated cells cultured in 3D and no contact with other cells, i.e., the cells cultured in media lacking FCS and kept in normoxia. Statistical significance was tested using non-parametric Kruskal-Wallis exact test and multiple comparison tests using GraphPad Prism version 5.0f, GraphPad Software, San Diego California USA, http://www.graphpad.com.

## Results

### Analysis of 2D and 3D cell populations

**Figure 3:**
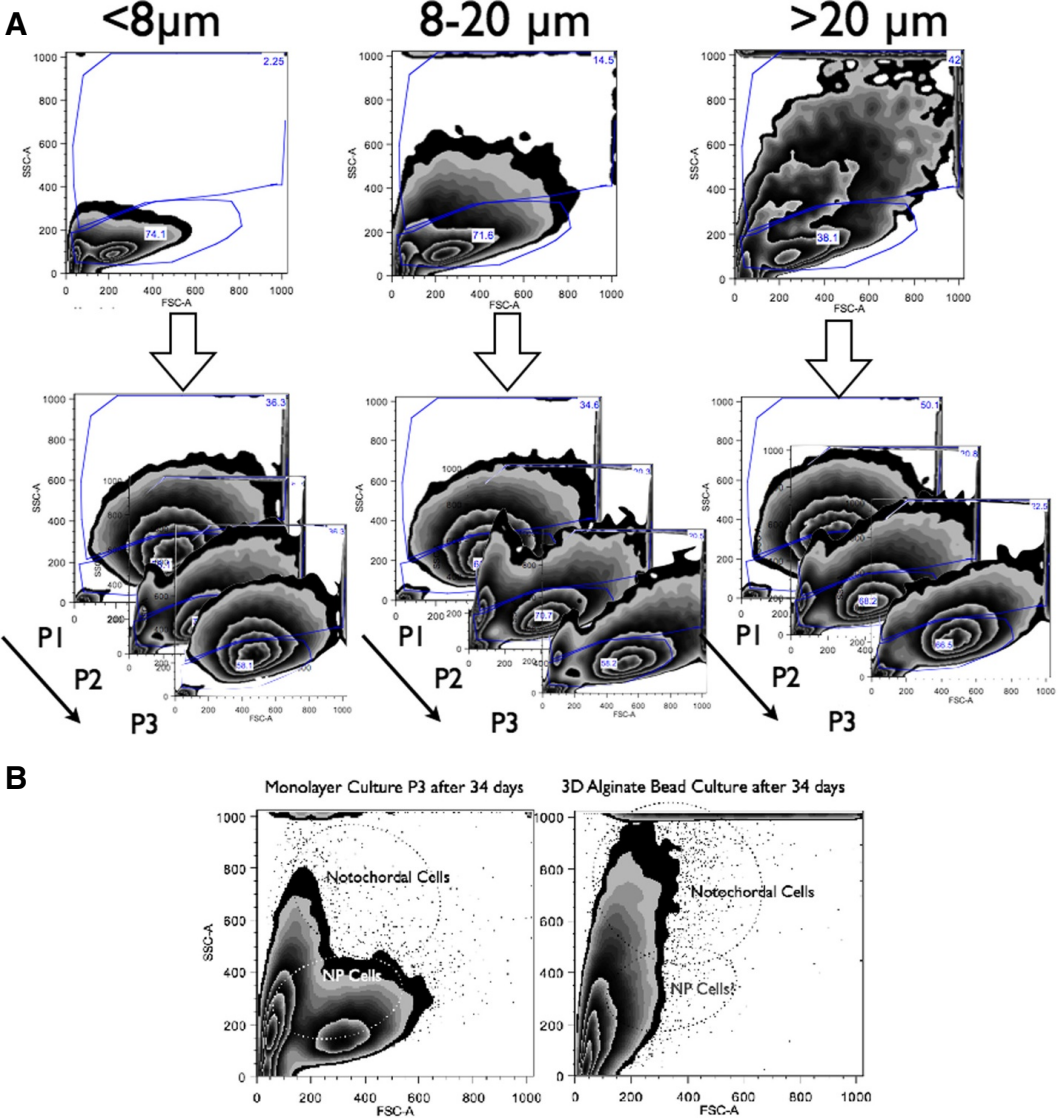
**Flow cytometry of cell-sieve sorted unlabeled primary cells isolated from porcine coccygeal intervertebral discs (IVD). A**. (first raw) flow cytometry of passage 0 IVD cells separated by size with cell sieves of different mesh size and (second raw) change of cell size and composition if IVD cells were kept in monolayer culture for three passages (after three weeks). **B**. Flow cytometry of unlabeled primary cells comparing the performance of 2D monolayer (lower left) versus 3D alginate culture (lower right). Parameters of flow cytometer were constant for all measurements. Axes are FSC-A = forward scatter, SSC-A = side scatter.

Cytometry was able to distinguish the cell size differences of the different cell types with the forward (F-SC) and side scatter (S-SC) (Figure 2). Initially putting cell isolations of different species unlabeled under the flow cytometer revealed the clear size differences of this primary isolated cell population after pronase/collagenase 2 digestion, with cell populations containing NC being much larger in F-SC and S-SC. This allowed the definition of the major gate for NC based on cell populations from the mixed IVD cell type. The gate for NPC was based on cells isolated from IVDs lacking the NC fraction, such as bovine and human IVD (Figure 2C, and D). Small NPC (< 8 μm cell size), a mixed cell population (8-20 μm), and a cell population with mainly large cells (>20 μm) were separated. Of the cells > 20 μm, almost all cells had a notochordal cell phenotype at the beginning of the culture. However, after 1-3 passages in 2D, the originally distinct cell populations turned into populations that were not distinguishable from each other (Figure 3A). In contrast, much more NC could be recovered after 34 days of 3D alginate bead culture (around 50%, see Figure 3B). Thus, 3D alginate culture seemed to be superior for NC culture as a higher fraction of large NC were revealed after 34 days of culture, compared to the identical cell population cultured in 2D monolayer and with the requirement to passage them three times for the same number of culture days (Figure 3).

### Cell metabolism, proliferation, and GAG synthesis of 3D co-culture experiments

**Figure 4:**
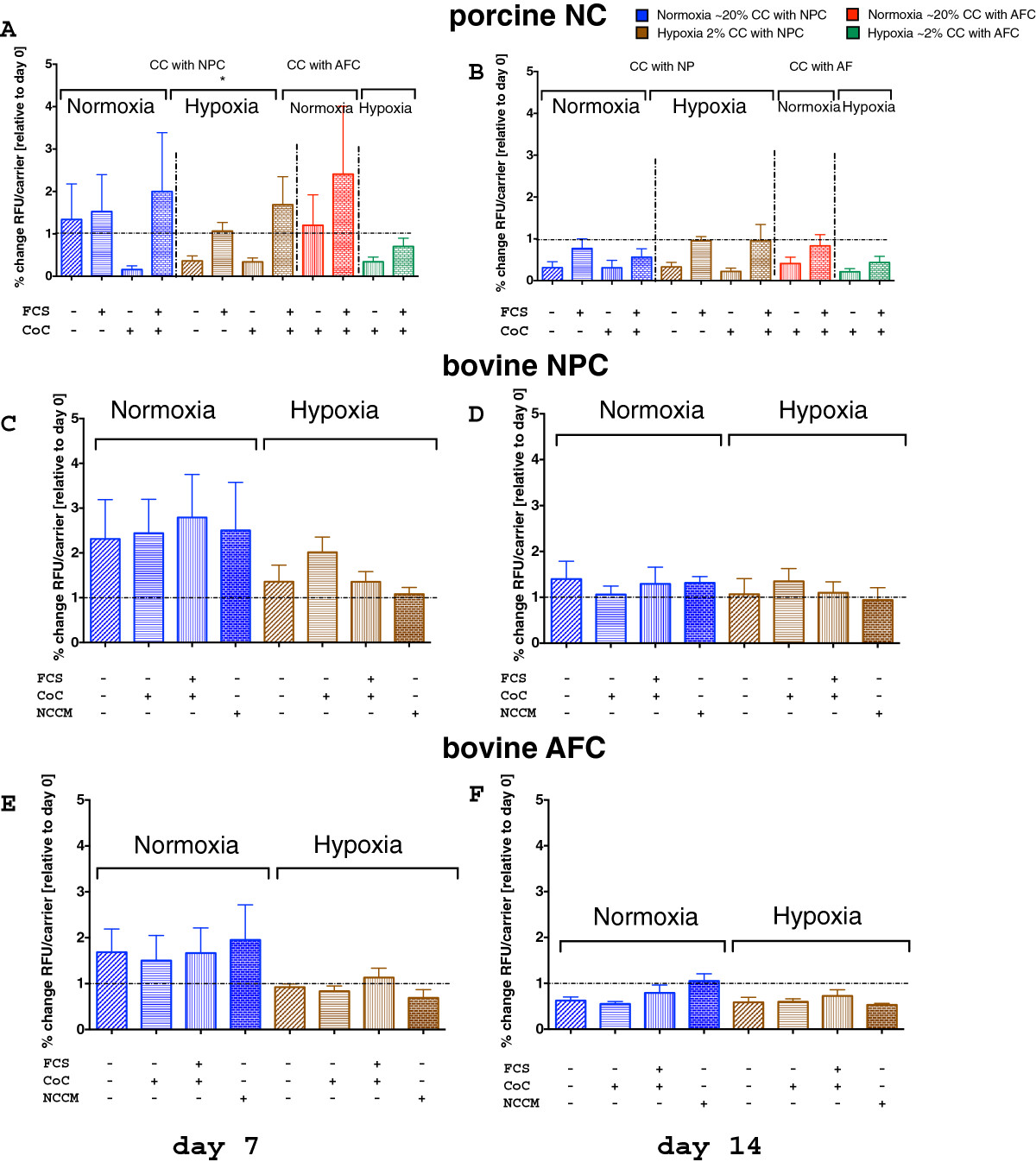
**Cell activity of primary cells in 3D alginate bead co-culture. A**, **B**. Porcine notochordal cells (NC) **C**, **D**. Bovine nucleus pulposus cells (NPC) and **E**, **F**. Bovine annulus fibrosus cells (AFC). The activity was measured by resazurin assay after day 7 (**A**, **C**, **E**, left side) and 14 days (**B**, **D**, **F**, right side) in normoxic (in blue) and hypoxic (in brown) conditions (mean ± SEM, N = 4 co-culture repeats). *Indicates p < 0.05.

**Figure 5:**
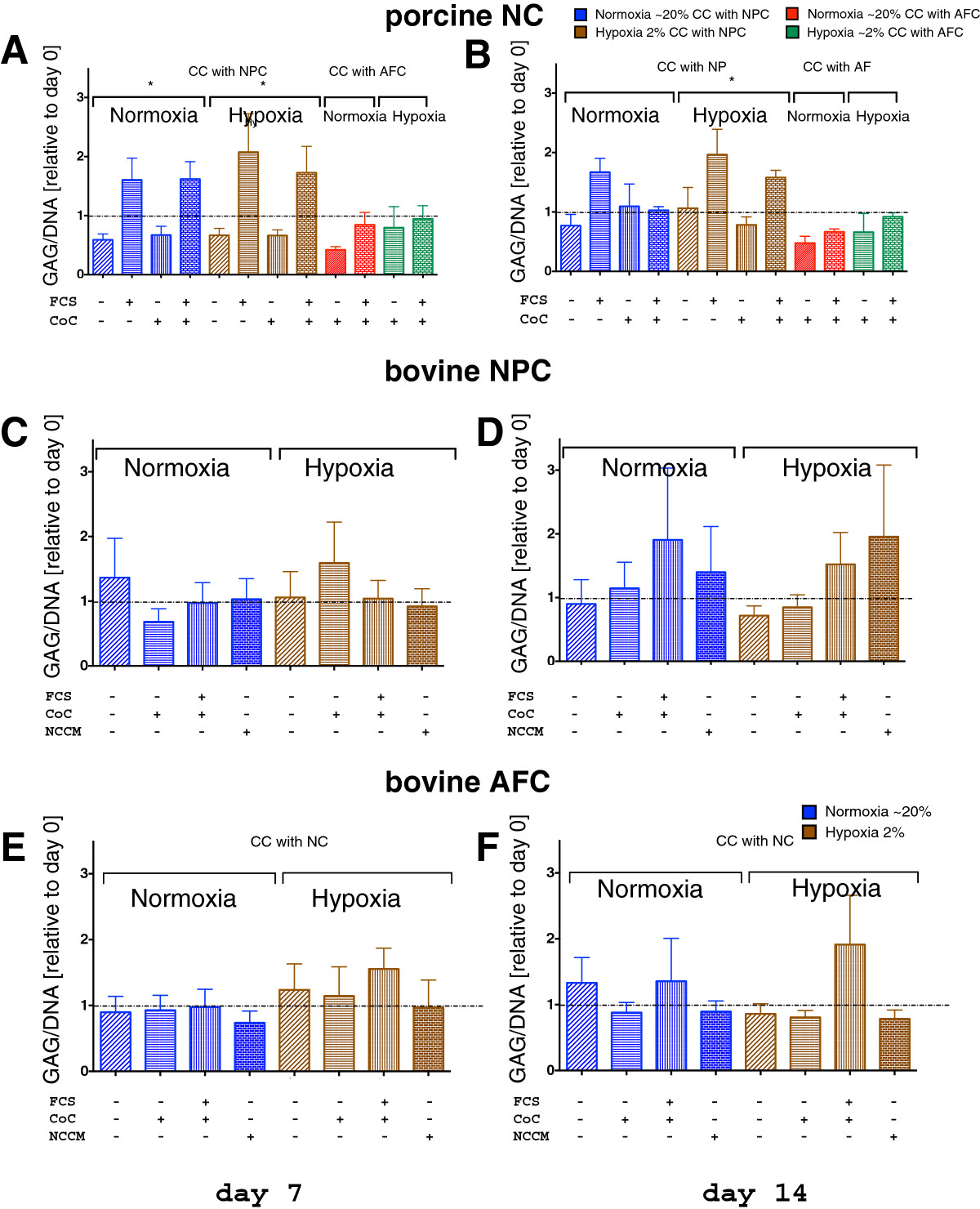
**GAG/DNA ratio of primary cells in 3D alginate bead co-culture. A**, **B**. Porcine notochordal cells (NC) **C**, **D**. Bovine nucleus pulposus cells (NPC) and **E**, **F**. Bovine annulus fibrosus cells (AFC). The ratio was measured after day 7 (**A**, **C**, **E**, left side) and 14 days (**B**, **D**, **F**, right side) of culture in normoxic (in blue) and hypoxic (in brown) conditions (mean ± SEM, N = 4 co-culture repeats). *Indicates p < 0.05.

After 7 days, cell activity, as quantified by resazurin assay, revealed a non-significant change among NC in normoxia (NO) among the four experimental groups (K-W, *p* = 0.117) but was significant among the experimental groups in hypoxia (HY) after 7 days (K-W, *p* = 0.0163, Figure 4A). After 14 days, activity was not different among NC groups, neither under NO nor HY (for NO K-W, *p* = 0.322, for HY K-W, *p* = 0.051) (Figure 4A and B). Pre-culture of NC prior co-culture revealed in general higher activity in the NC than in the controls after 7 days of co-culture. However, these effects flattened out after 14 days. NPC cell activity remained unchanged under NO after 7 days. There were no significant differences in cell activity among the NPC and the AFC experimental groups, whether they were cultured in NO or HY (Figure 4E and F). The GAG/DNA ratio among the NC groups was significantly different after 7 days in both NO (K-W, *p* = 0.023, Figure 5A) and HY conditions (K-W, *p* = 0.016 for HY, Figure 5A), and the ratio remained significantly different after 14 days in HY (K-W, *p* = 0.044, Figure 5B). In NO conditions, the GAG/DNA ratio of pure NPC was decreased on day 14 to 89.9 ± 38.3% (mean ± SEM) (Figure 5D). When these NPC were co-cultured with NC 1:1, which were never exposed to FCS, the GAG/DNA ratio dropped after 7 days (i.e. 77.1 ± 18.68%, Figure 5C). However, in NO, if we added NCCM or co-cultured NPC with NC, which were exposed to 10% FCS during pre-culture, the ratio tended to be increased (190.6 ± 112.8%) or by addition of NCCM (140.1 ± 71.5%, Figure 5D) after 14 days. However, this was statistically non-significant among NPC groups (K-W, *p* = 0.964). In HY, however, the ratio tended to be elevated to 152.1 ± 50.2% for the CoC and to 195.7% ± 112.5% for the NCCM group (Figure 5D). After 14 days of culture, these trends were maintained, but these were not significant.

### Relative gene expression of NC

**Figure 6:**
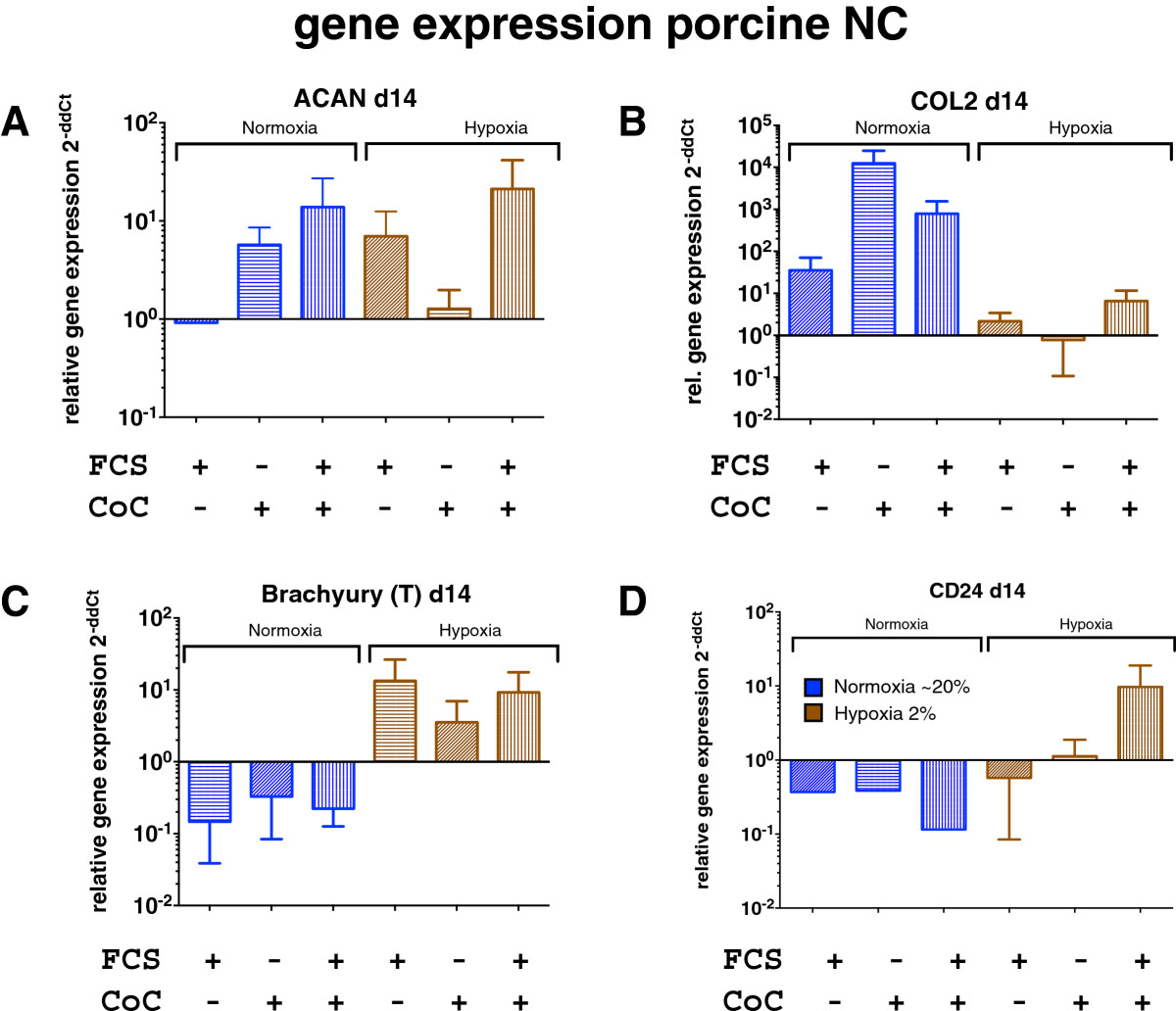
**Relative gene expression of porcine NC in co-culture with bovine NPC in 3D alginate microspheres after 14 days of culture in normoxic (in blue) and hypoxic conditions (in brown). A**. Aggrecan (ACAN) expression, **B**. Collagen type 2 (col2) expression, **C**. Brachyury (T) expression, **D**. CD24 expression *indicates p < 0.05. Gene expression is given relative to the pure NC without FCS and in normoxia (NO) at 14 days. CoC = co-culture with NPC, FCS = 7 days pre-culture in medium containing 10% fetal calf serum (mean ± SEM, N = 4 co-culture repeats).

ACAN expression of NC was found up-regulated around ten times in the co-culture groups with NPC and previous exposure to FCS, compared to the pure cell population without previous contact with FCS, both in NO and HY (Figure 6A). Further, we found that ACAN gene expression were more pronounced in the co-cultures supplied with 10% FCS and were more strongly expressed in the NO cultures (Figure 6). Collagen type 2 expression was up-regulated much stronger in all groups cultured in NO but much less in HY. The gene expression of one of the major genes for the characterization of the notochordal cells – brachyury (T) — showed a weak up-regulation after 14 days of culture in the HY group, but not in the NO group (Figure 6C).

### Relative gene expression of NPC

**Figure 7:**
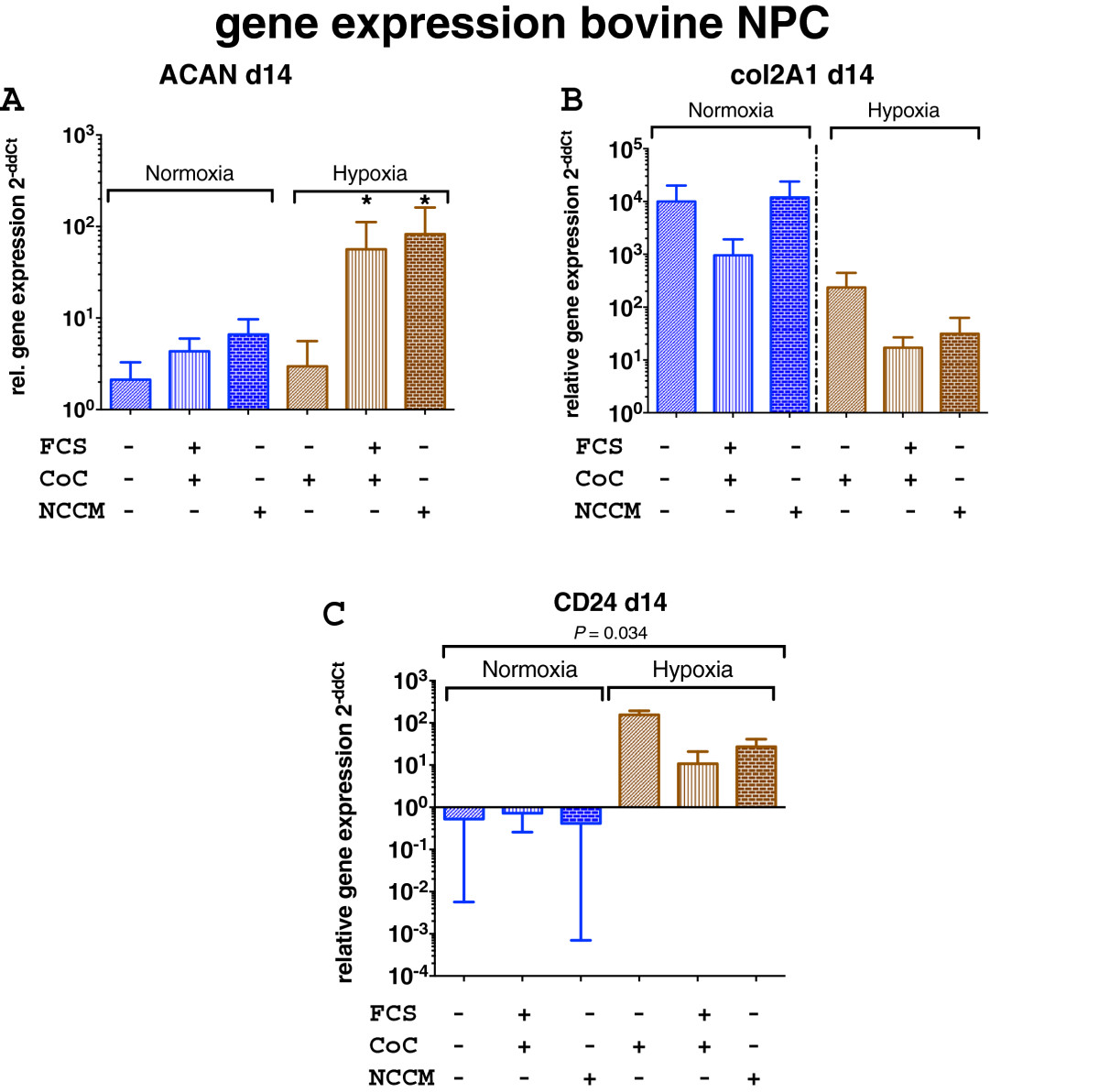
**Relative gene expression of bovine NPC in co-culture of bovine NC in 3D alginate microspheres after 14 days of culture in normoxic (in blue) and hypoxic (in brown) conditions. A**. Aggrecan (ACAN) expression, **B**. Collagen type 2 (col2) expression, **C**. CD24 expression. *Indicates p < 0.05. Gene expression is given relative to the pure NPC without FCS and in normoxia (NO) at 14 days, CoC = co-culture with NC, FCS = 7 days pre-culture in medium containing 10% fetal calf serum (mean ± SEM, N = 4 co-culture repeats).

**Figure 8:**
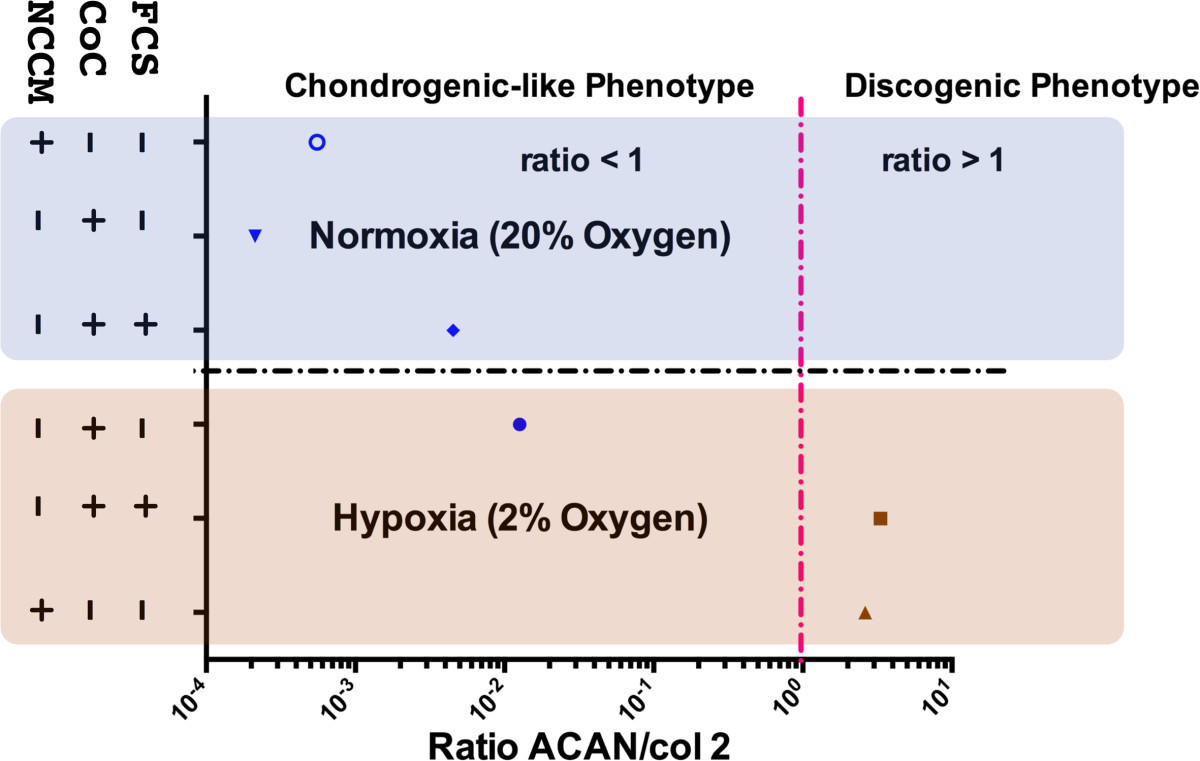
**ACAN/col2 ratio of relative gene expression of bovine nucleus pulposus cells (NPC) cultured for 14 days either in normoxia (NO = 20% oxygen) or hypoxia (HY = 2% oxygen).** NCCM = porcine notochordal cells conditioned medium, CoC = co-culture with NC, FCS = 7 days pre-culture in medium containing 10% fetal calf serum.

RT-PCR data suggested that CD24 expression in bovine NPC (Figure 7), the NP-specific surface marker, was expressed differently in NO and HY conditions. While there was only a weak effect between the experimental groups of co-culture, however, the effect of the oxygen level was significant (K-W, p = 0.034). However, according to the ACAN/col2 ratio criterion, the most “IVD-like” gene expression pattern among all investigated NPC groups was identified in HY and by co-culture with NC that were previously activated with FCS or by addition of NCCM (Figure 8).

### Histology

**Figure 9:**
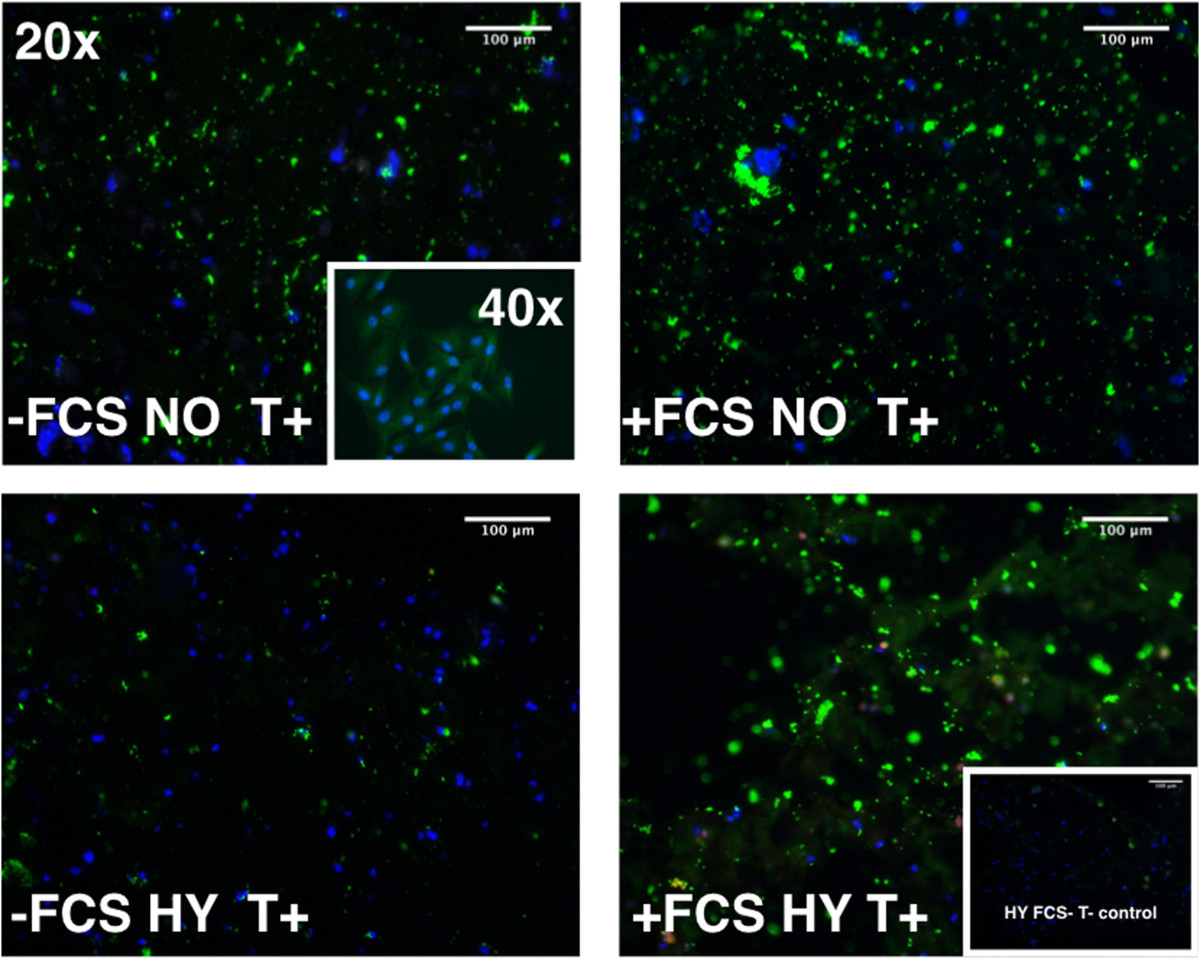
**Immunological staining of brachyury (“T”, green) expression on porcine NC in alginate bead cryosections (12** **μm) and DAPI stain for nuclei after 14 days of culture in presence or absence of initial pre-culture with 10% fetal calf serum (FCS) and cultured either in normoxia (~20% oxygen) or hypoxia (2% oxygen).** Inlet in the upper left represents day 4 porcine NC cultured on cover slips in monolayer and stained for T. Inlet in the lower right represents a negative control lacking the primary antibody.

The immune histology of NC in beads for brachyury (T) revealed a slightly stronger expression in the case of NO culture conditions compared to HY but the strongest effect was seen if NC were kept “boosted” in media containing 10% FCS for 7 days previous to co-culture (Figure 9).

### Mass spectrometry of NCCM

For the analysis of the medium protein profile (Table 2, Additional file1: Table S1), we screened for strong signal differences between the unconditioned and the conditioned medium (pooled after 7 days of serum-free culture). A full table of the Swiss Prot search of identified proteins can be obtained as an additional supplemental, Additional file1: Table S1. We found that the CTGF increased in the conditioned medium, which has been identified in canine notochordal cells by Erwin et al.[24]. Further, we found a high concentration of the serum amyloid A-2 protein (SAA2_MUSVI), an outbreak protein identified in immune burst reactions (Table 2 and Additional file1: Table S1).

**Table 2:** **Summary of mass spectrometric analysis of the most interesting proteins related to the IVD extracellular matrix homeostasis identified in serum free medium (SFM) on day 0, conditioned medium (NCCM) of pure NC collected after 7 days and co-culture (CoC) medium of 1:1 NC: NPC co-culture (CoC) after 7 days CoC**

ID	Full name	SFM	NCCM	CoC 1:1	cnts
***ECM***					
CO1A1_BOVIN	collagen type 1 A1 chain	20.0	**46.4**	**33.6**	3
CO1A2_BOVIN	collagen type 1 A2 chain	23.4	**40.1**	**36.5**	3
CO5A1_HUMAN	Collagen alpha-1(V) chain	24.0	**40.0**	**36.0**	3
CO6_BOVIN	Collagen alpha-1(VI) chain	**57.1**	**42.9**	0.0	2
COMP_HUMAN	Cartilage oligomeric matrix protein (COMP)	26.8	**36.6**	**36.6**	3
***Cytokines***					
CTGF_PIG	Connective tissue growth factor	0.0	**40.0**	**60.0**	2
CXL16_PIG	C-X-C motif chemokine 16	0.0	0.0	**100.0**	1
GALA_BOVIN	Galanin message-associated peptide (GMAP)	0.0	0.0	**100.0**	1
IBP2_PIG	Insulin-like growth factor-binding protein 2 (IBP-2)	0.0	**61.8**	**38.2**	2
IBP5_HUMAN	Insulin-like growth factor-binding protein 5 (IBP-5)	6.8	0.0	**93.2**	2
IL6_BOVIN	Interleukin-6 (IL-6) [CHAIN 0]	0.0	0.0	**100.0**	1
***Metalloproteinases***					
MMP1_HORSE	Interstitial collagenase (MMP-1)	0.0	**100.0**	0.0	1
MMP1_PIG	18 kDa interstitial collagenase	0.0	**86.5**	13.5	2
MMP2_BOVIN	PEX	0.0	0.0	**100.0**	1
***Inhibitors of Metalloproteinases***					
TIMP1_BOVIN	Metalloproteinase inhibitor 1 (EG-1, TIMP-1)	0.0	0.0	**100.0**	1
TIMP1_PIG	Metalloproteinase inhibitor 1 (TIMP-1)	0.0	**47.4**	**52.6**	2
***Proteases***					
HTRA1_HUMAN	Serine protease HTRA1	0.0	0.0	**100.0**	1
***Other***					
ALBU_PIG	Serum albumin	0.0	**59.6**	**40.4**	2
PRDX1_HUMAN	Peroxiredoxin-1 (NKEF-A) (PAG)	0.0	**100.0**	0.0	1
PRG4_HUMAN	Proteoglycan 4 C-terminal part [ISOFORM E]	0.0	0.0	**100.0**	1
SAA1_MUSVI	Serum amyloid A-1 protein	0.0	**100.0**	0.0	1
SAA2_MUSVI	Amyloid protein A [CHAIN 0]	0.0	**78.0**	22.0	2
CLC11_HUMAN	C-type lectin domain family 11 member A	0.0	0.0	**100.0**	1

SFM = serum free medium control, CoC 1:1 = NPC: NC Co-Culture, NCCM = Conditioned Medium.

The numbers indicate peptide spectrum matches, expressed as percentage from the total over all three media. Cells were kept under normoxia.

Number in bold are percentages > 33%.

## Discussion

### Phenotype of NC

The NC phenotype is specific in the sense that cells are relatively large in size[47] and highly dependent on nutrition because of a high cellular activity[23, 28]. Here, we present data that clearly demonstrate that in monolayer cell culture, NC seem to be outcompeted by the small chondrocyte-like NPC (Figure 3). Of course, we cannot conclude from our unlabeled FACS cytometric data whether the NC differentiated into NPC or whether NPC simply grew faster than NC. However, a recent time-lapse microscopy study by Kim et al.[48] showed that NC populations harvested from the spine of mature New Zealand white rabbits proliferate much slower than the smaller chondrocyte-like cells (i.e. defined as NPC in our case). These authors, furthermore, demonstrated, using time-lapse cell-tracking microscopy, that by looking closer into the NC fraction, there are different cell types present, i.e., vacuolated, giant and polygonal cells that differ in cell proliferation and speed of cell motility. These authors suggested that NC differentiated in vitro into these three morphologically different cell types. The negative effects of monolayer culture for the maintenance for NC has also been demonstrated by Rastogi et al.[26], who cultured rat NC in monolayer in NO and HY. They found that 3D alginate culture maintained the NC phenotype better compared to 2D monolayer. The rapid loss of NC over time can be partially prevented by 3D alginate culture for up to 34 days, as was revealed in this study (Figure 3). Considering our data and the published work, it is still unclear which scenario might be true. We further confirm a strong nutrient dependency (by addition or lack of FCS), as has been found previously[28]. We can clearly confirm a strong dependency of NC to culture medium composition and also oxygen content, as revealed by cell activity and GAG/DNA ratio data (Figures 4 and5). We found a trend towards increased GAG/DNA ratio if NC were kept in medium containing 10% FCS (Figure 5). It has been demonstrated that NC and NPC differ in cell size, nutrition[28], surface markers[49], and mechanosensitivity[29]. It was found that NC glucose consumption rate is higher than in NPC under identical culture conditions[28]. Furthermore, the clear size differences were confirmed by non-invasive femtosecond laser microscopy[47]. Furthermore, the NC differ by the presence of large vacuoles, which can be separated by the size-scatter of FACS analysis[3, 6], as has also been shown by our study. These large vacuoles found in NC of the intervertebral disc have been attributed to a possible functional role in osmoregulation[50]. It has been noted that these vacuoles are not present anymore in the NC after long-time in-vitro culture of 28 days using porcine NC[27, 51]. No such complete loss of vacuoles but a reduction in numbers were reported by other in vitro studies using rat or rabbit NC[26, 48]. Transcriptomics that compare the NC and the NPC cell population revealed that there are only about 20 genes really distinct between these two cell types[10, 52]. A number of cell surface markers that separate these two cell populations have been identified[49]. Weiler et al.[53] found that cells in the human fetal and juvenile NP with the typical morphology of the notochord (physaliferous) express the markers cytokeratin CK-8, -18, -19, and Galectin-3[54]. Another NC-typical marker is brachyury (T), which was investigated in this study. We found that the notochordal homeobox gene marker T did not change when altering the culture conditions but decreased about 10-fold under normoxia at the RNA level (Figure 6). Immunohistology staining confirmed this finding and showed a slight increase of T expression in the case of hypoxia (Figure 9). Potier et al.[27] found a general loss of the NC phenotype and loss of the markers cytokeratin 8 and 19 (KRT8, 19) in their co-culture system of NC, NPC, and mesenchymal stem cells over 28 days of culture. Gilson et al.[49] found that pig NPC, which are phenotypically similar to human infant NPC, were all KRT8 positive. This has been recently confirmed by another histological study on human tissue; the authors furthermore demonstrated that KRT8 is down-regulated with onset of IVD degeneration[55]. However, as we did not look into KRT8 expression in this study, we cannot comment on the validity of the KRT8 marker. Interestingly, a strong effect between hypoxic and normoxic condition was found in the surface marker CD24 in the NPC (K-W test, *p* = 0.034) (Figure 7), such that CD24 was higher expressed under HY. This finding is in agreement with the culture study presented by Rastogi et al.[26] where they kept NC in alginate beads and in hypoxia and normoxia. Thus, CD24 might indeed represent a sensitive marker for the NC phenotype. CD24, a glycosylphosphatidylinositol (GPI) anchor protein, has been proposed as a NP-specific marker[26, 41]. Finally, a Tie2+ cell population has been recently reported about, which reflects a stem cell population in the IVD, which decreases rapidly with age[56]. These cells, as well as induced progenitor (iPS) cells, seem to express CD24 and can be enriched via this surface marker[16, 56]. iPS cells, which hold potential for the regeneration of the IVD, could at least theoretically be produced from both NC and NPC. However, it is still unclear which cell type would be more potent and suitable.

### Optimizing NC culture conditions

NC are perfectly adapted to a low oxygen environment. They can produce a better-aligned ECM under hypoxic conditions[20]. Here, we cultured the cells under normoxic and hypoxic conditions. We found that the GAG/DNA ratio of the NC could be significantly increased indeed under HY rather than NO after 14 days (Figure 5B). However, in terms of the better maintenance of a notochord phenotype, the expression of T was higher under HY than in NO from both gene expression and immune-staining data. It could also be that keeping the NC in clusters rather than completely isolated by digestion and increasing the osmolarity from 230 mOsm/L to 400 mOsm/L could preserve the porcine NC phenotype better in culture[51]. Interestingly, we found that by applying indirect co-culture or applying the CM to the bovine NPC, we could obtain a ACAN/col 2 ratio, which was most favorable for the IVD phenotype, in the order of a 1:1 ratio as found in rabbit cells[32] (Figure 8). Similar findings were obtained by previous studies culturing NC or NPC under hypoxia[16, 20, 31, 57]. We cultured the NC in high glucose DMEM (i.e. with 25 mM glucose) either in serum-free medium or medium supplemented with 10% FCS. Park and Park[58] found that the cell proliferation of NC was significantly decreased at glucose concentrations of 100 mM, 200 mM, and 400 mM and apoptosis was increased via the intrinsic pathway with dose- and time-dependent effects. Guehring et al.[28] tested the nutrient sensitivity of NC depleting glucose from 5 mM to 0 mM and found a strong nutrient sensitivity of these cells. Future experiments could be to test culture conditions on NC viability in the 5-20 mM range and adjusting osmolarity.

### Composition of NCCM

The analysis of the NCCM by mass spectrometry revealed the presence of expected anabolic proteins, such as the collagens 1, 5, and 6, which these cells produce in 2D and surely in 3D culture (Table 2). Notably, collagen type 2 was not hit by the SwissProt blast. There were also two MMPs detected (MMP1 and MMP2) detected, which is not unexpected given the nature of the NC and NPC. However, unexpectedly, neither MMP3 nor MMP13 were hit by the SwissProt search in the NCCM. These missed proteins might be due to the limited approach to analyzing the NCCM and CoC-medium presented here. Future experimental work could specifically isolate the vacuoles of the NC through centrifugation methods and then perform mass spectrometry analysis on these fractions, which might reveal more focused spectrum searches. Another interesting candidate found by the SwissProt search was High-Temperature Requirement A Serine Peptidase (HTRA1) that was present in the case of the 7 days CoC of NC with NPC (Table 2, Additional file1: Table S1). This enzyme has been described as a potential key enzyme in intervertebral disc degeneration[59, 60] and was specifically expressed here by NPC and not by NC.

## Conclusions

- NC were sensitive to the addition of FCS to the culture medium after 7 days of co-culture but these effects were not longer evident after 14 days. The cell activity was highly reduced in hypoxia compared to normoxia (Figure 4A and B).
- NC produced significantly more GAG/DNA after stimulation with 10% FCS in NO and HY after 7 days culture and in HY after 14 days culture (Figure 5A and B).
- NPC tended to produce more GAG/DNA in presence of NC that have been activated previously with FCS or by addition of NCCM (Figure 5C and D).
- AFC, however, did not respond at all to the presence of NC nor NCCM (Figure 5E and F).
- Hypoxia and indirect co-culture of FCS-stimulated NC or addition of NCCM resulted in a NPC phenotype, which was most IVD-like according to the ACAN/col2 ratio (Figure 8).
- Connective Tissue Growth factor (CTGF, syn. CCN2) has been confirmed to be present in the conditioned medium of porcine NC and in NC: NPC co-cultures (Table 2).

## Electronic supplementary material

Additional file 1: Table S1: Mass spectrometric comparison between serum free medium (SFM), co-culture medium of 1:1 NC: NPC co-culture (CoC) (collected after 7 days) and conditioned medium (NCCM) of pure NC. The numbers indicate peptide spectrum matches and sequence coverage. (XLS 410 KB)

Below are the links to the authors’ original submitted files for images.

Authors’ original file for figure 1

Authors’ original file for figure 2

Authors’ original file for figure 3

Authors’ original file for figure 4

Authors’ original file for figure 5

Authors’ original file for figure 6

Authors’ original file for figure 7

Authors’ original file for figure 8

Authors’ original file for figure 9

## Abbreviations

AFC: Annulus fibrosus cells
COC: Co-culture
FCS: Fetal calf serum
KRT: Keratin
MMP: Metalloproteinase
NO: Normoxia
NCCM: Notochordal cell-derived conditioned medium
NPC: Nucleus pulposus cells
HY: Hypoxia
pNC: Porcine notochordal cells.
