# Supplementary material for: Activation of intervertebral disc cells by co-culture with notochordal cells, conditioned medium and hypoxia
Source: BMC Musculoskelet Disord. 2014 Dec 11;15:422. doi: 10.1186/1471-2474-15-422 (PMC4295479; doi:10.1186/1471-2474-15-422)
Supplement: Supplementary file 2 — Authors’ original file for figure 1 [file 12891_2014_2382_MOESM2_ESM.pdf]

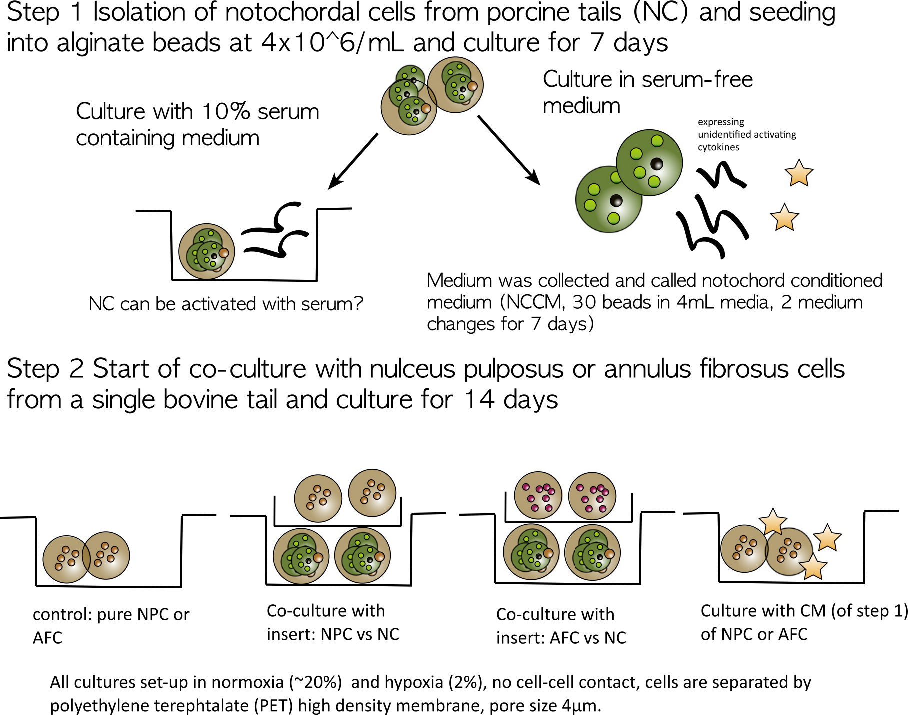

Step 1 Isolation of notochordal cells from porcine tails (NC) and seeding into alginate beads at 4x10^6/mL and culture for 7 days
Culture with 10% serum containing medium
Culture in serum-free medium
expressing unidentified activating cytokines
NC can be activated with serum?
Medium was collected and called notochord conditioned medium (NCCM, 30 beads in 4mL media, 2 medium changes for 7 days)
Step 2 Start of co-culture with nulceus pulposus or annulus fibrosus cells from a single bovine tail and culture for 14 days
control: pure NPC or AFC
Co-culture with insert: NPC vs NC
Co-culture with insert: AFC vs NC
Culture with CM (of step 1) of NPC or AFC
All cultures set-up in normoxia (~20%) and hypoxia (2%), no cell-cell contact, cells are separated by polyethylene terephtalate (PET) high density membrane, pore size 4µm.
