# Supplementary figures and images for: Activation of intervertebral disc cells by co-culture with notochordal cells, conditioned medium and hypoxia
Source: BMC Musculoskelet Disord. 2014 Dec 11;15:422. doi: 10.1186/1471-2474-15-422 (PMC4295479; doi:10.1186/1471-2474-15-422)

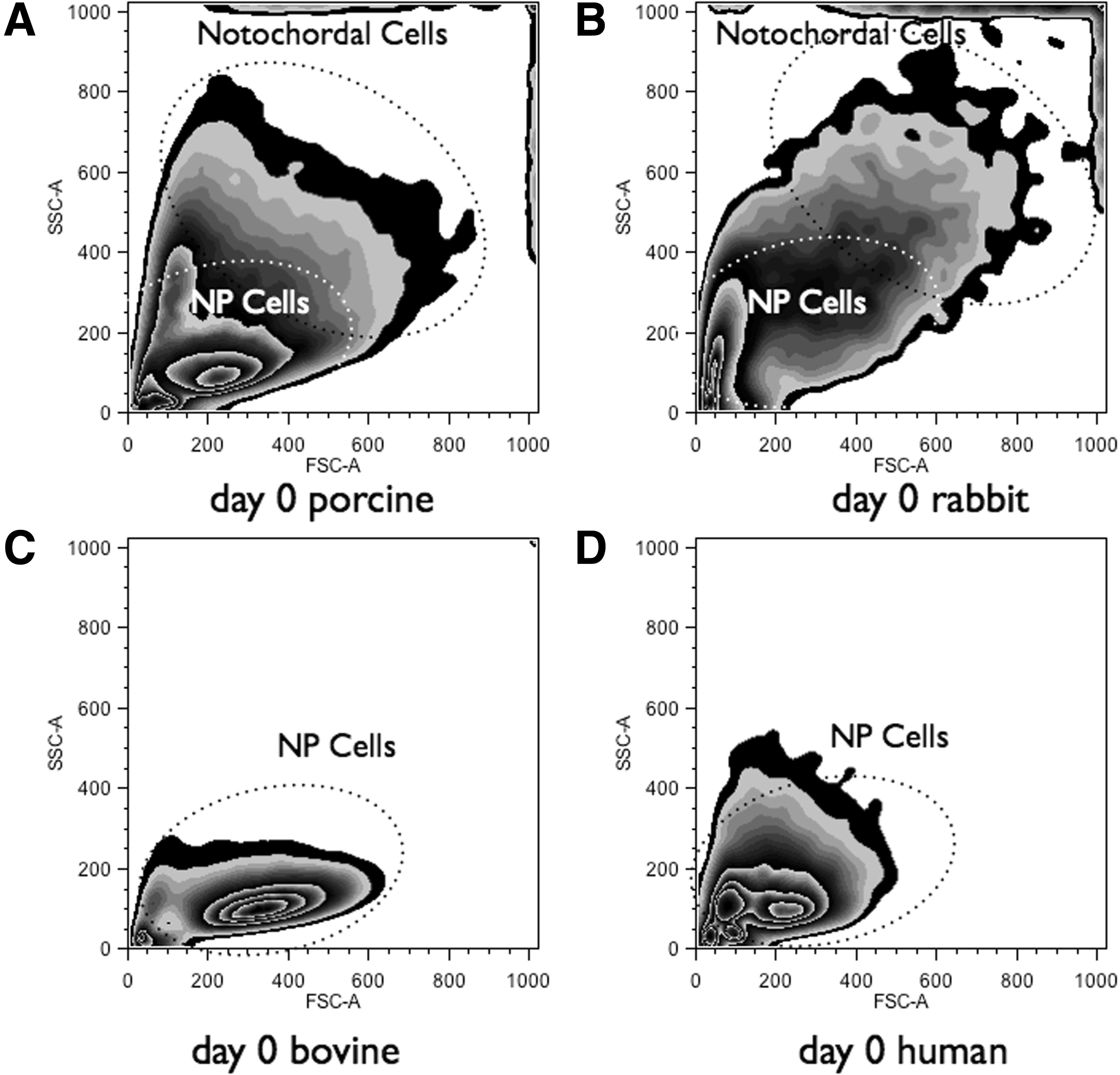

Supplement: Supplementary file 3 — Authors’ original file for figure 2 [file 12891_2014_2382_MOESM3_ESM.tif]

**A** <8 $\mu$ m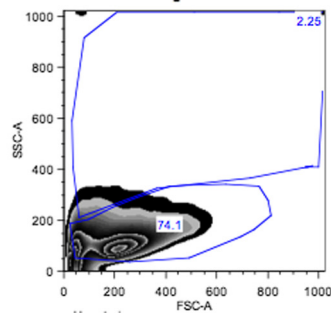8-20  $\mu$ m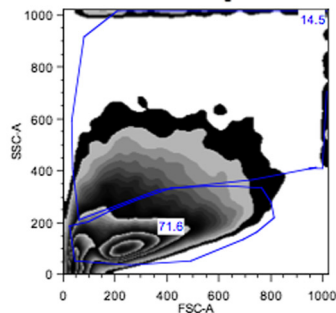>20  $\mu$ m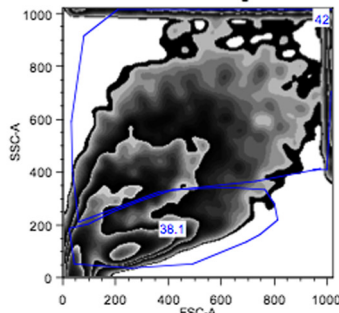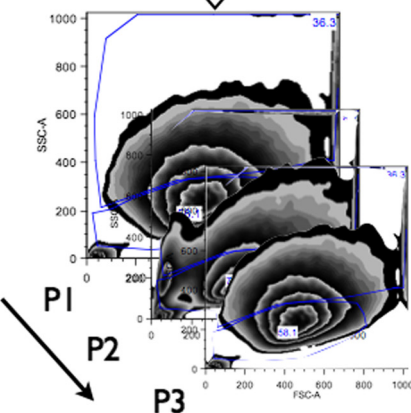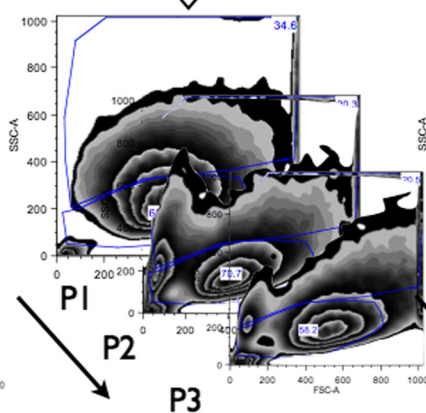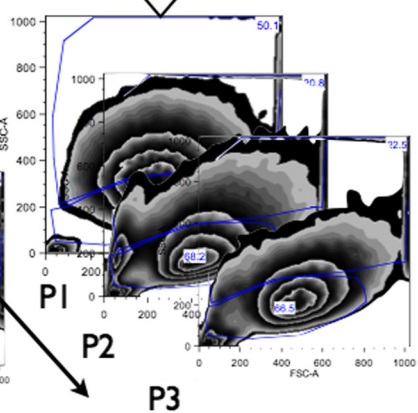**B**

Monolayer Culture P3 after 34 days

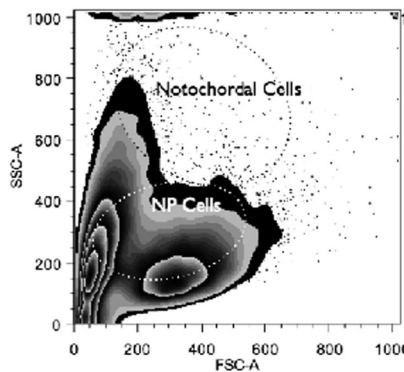

3D Alginate Bead Culture after 34 days

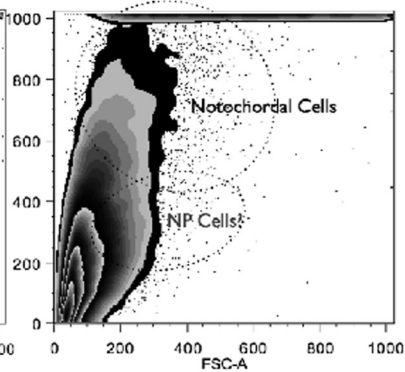

Supplement: Supplementary file 4 — Authors’ original file for figure 3 [file 12891_2014_2382_MOESM4_ESM.pdf]

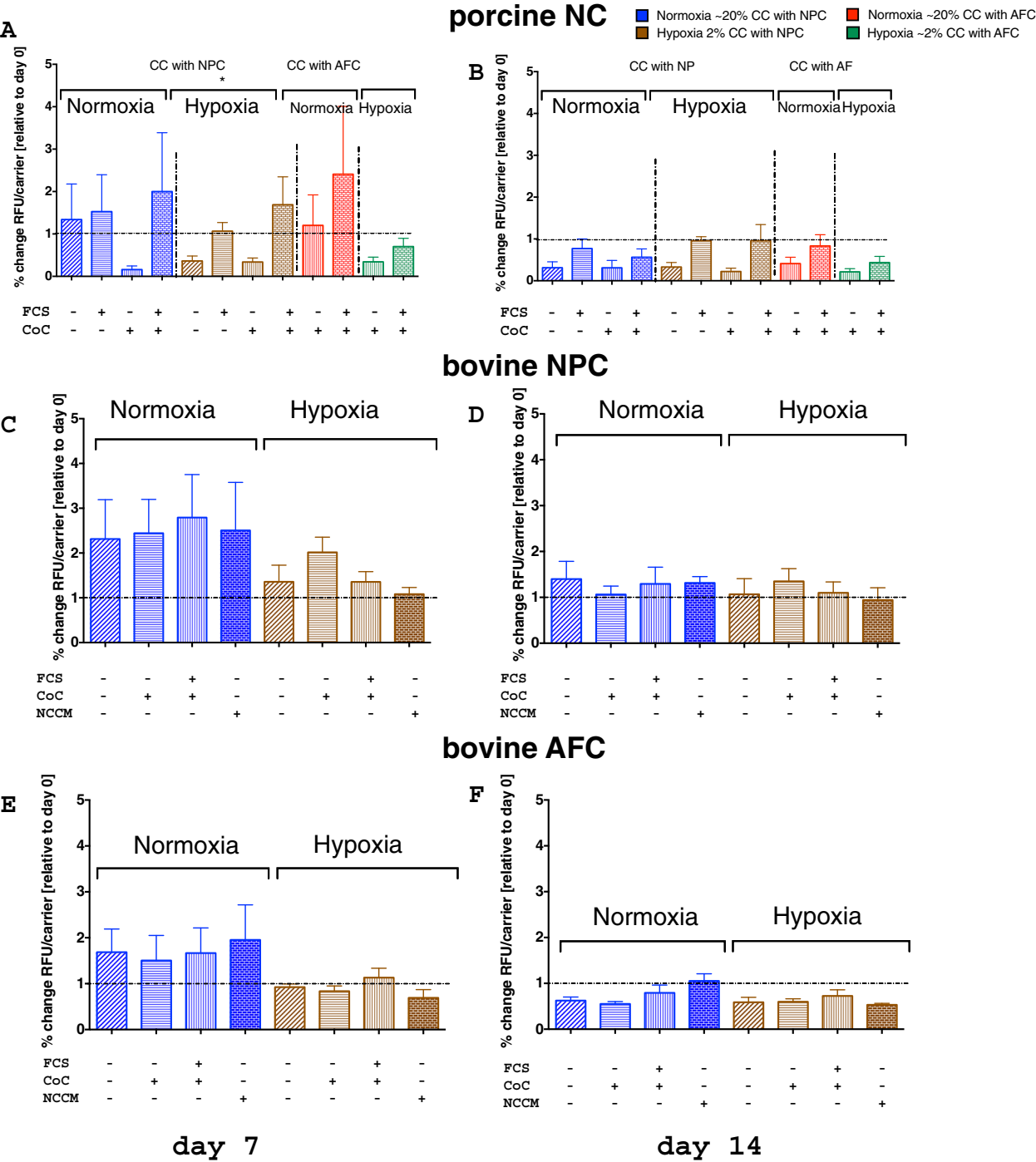

Supplement: Supplementary file 5 — Authors’ original file for figure 4 [file 12891_2014_2382_MOESM5_ESM.pdf]

# porcine NC

**A**

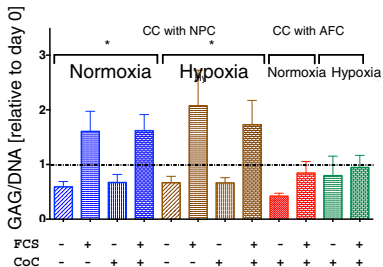

**B**

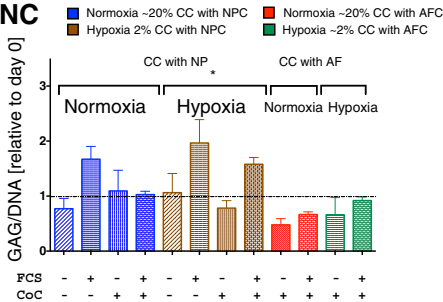

# bovine NPC

**C**

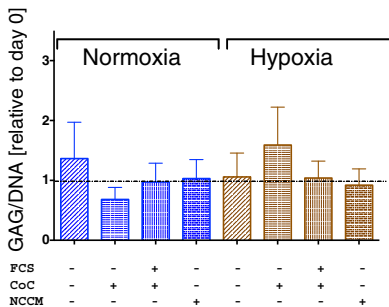

**D**

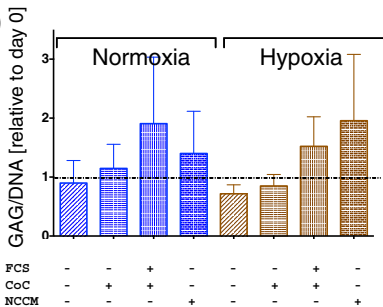

# bovine AFC

**E**

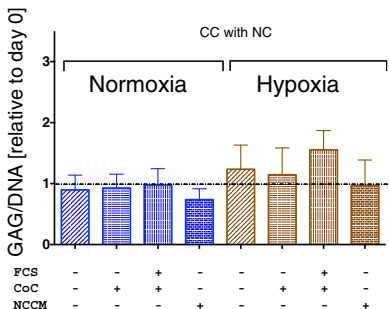

**F**

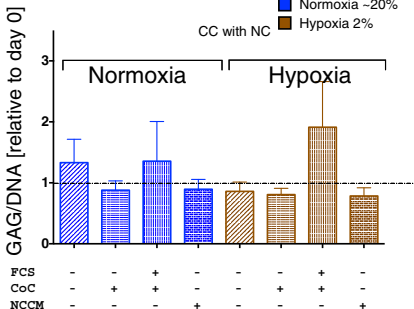

day 7

day 14

Supplement: Supplementary file 6 — Authors’ original file for figure 5 [file 12891_2014_2382_MOESM6_ESM.pdf]

# gene expression porcine NC

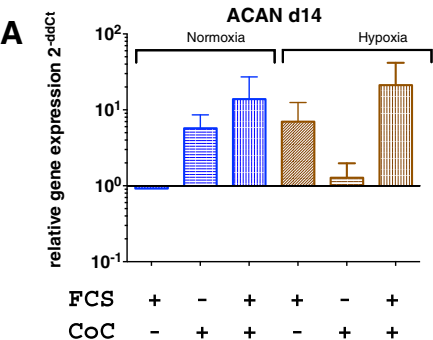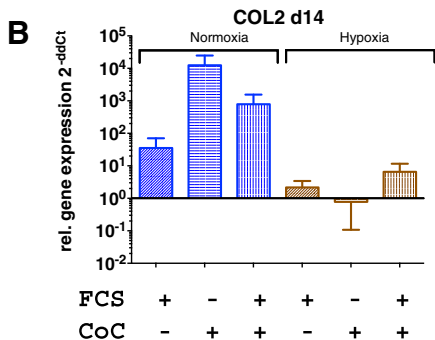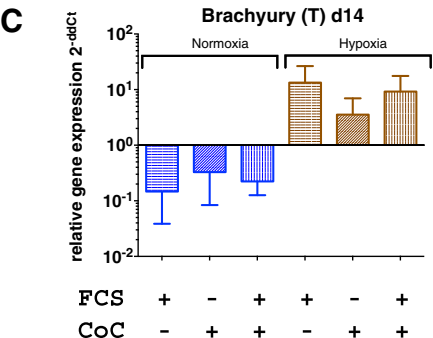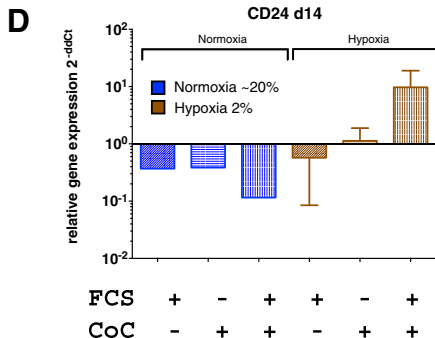

Supplement: Supplementary file 7 — Authors’ original file for figure 6 [file 12891_2014_2382_MOESM7_ESM.pdf]

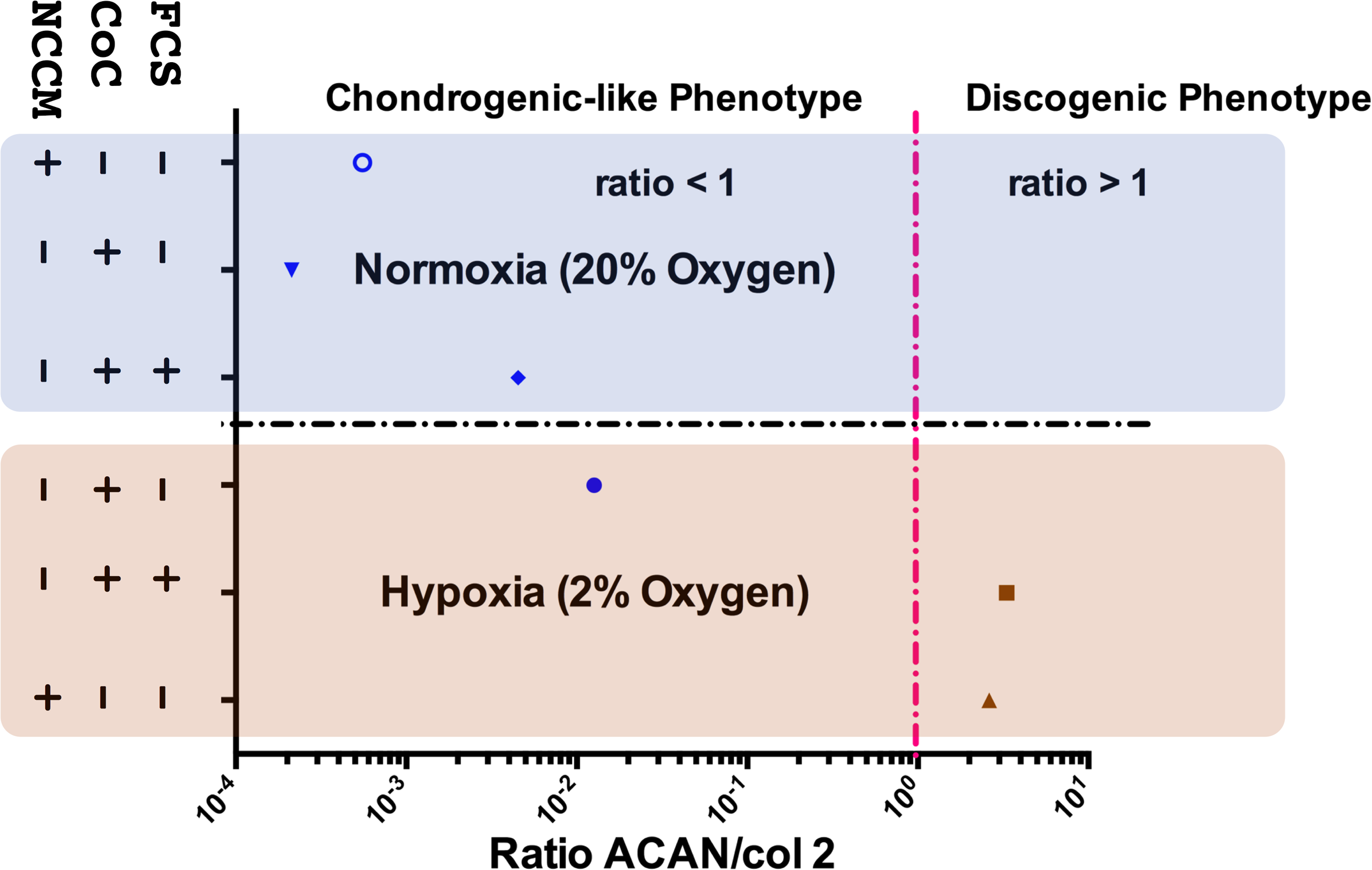

Supplement: Supplementary file 9 — Authors’ original file for figure 8 [file 12891_2014_2382_MOESM9_ESM.tif]

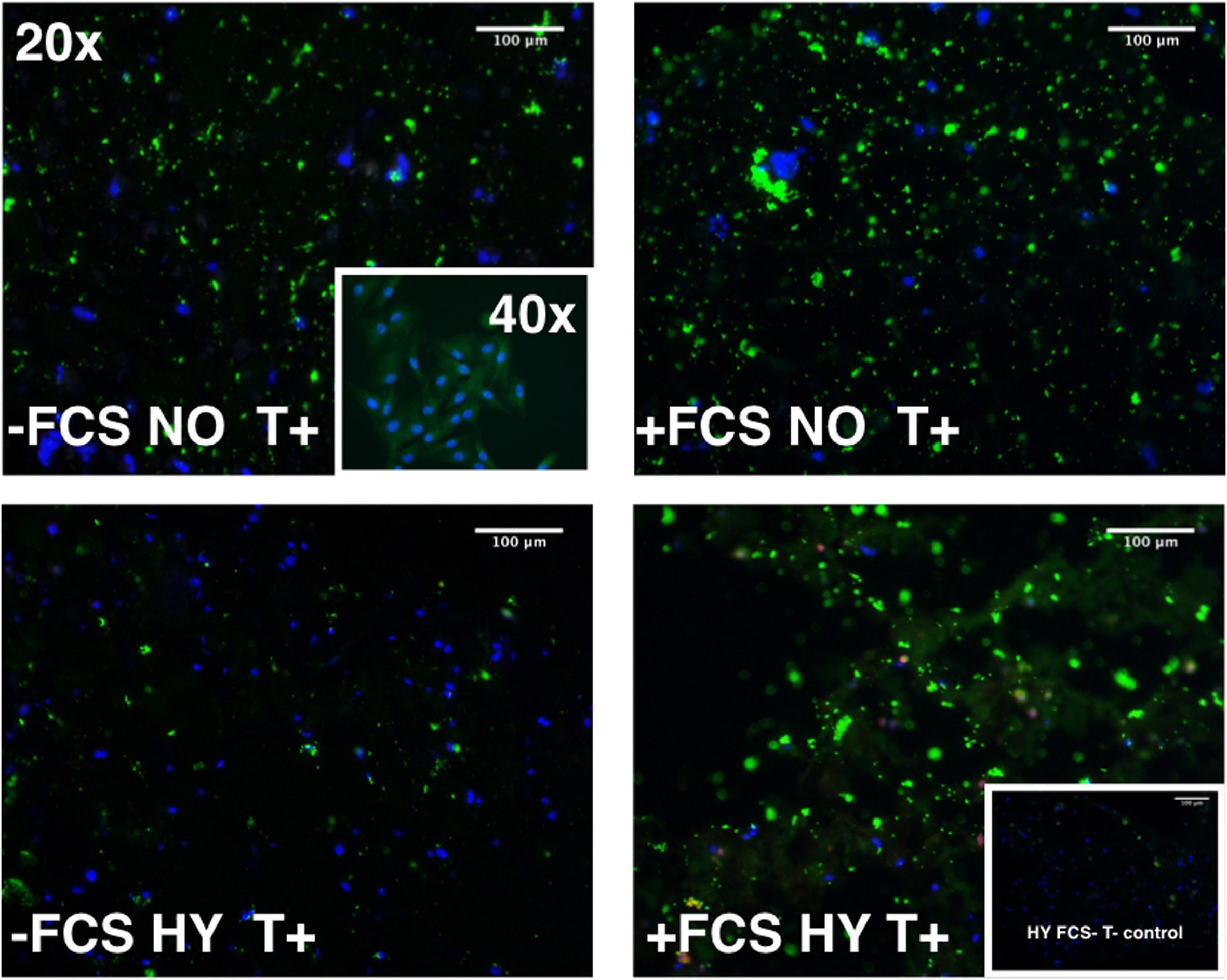

Supplement: Supplementary file 10 — Authors’ original file for figure 9 [file 12891_2014_2382_MOESM10_ESM.tif]
