# Supplementary material for: Activation of intervertebral disc cells by co-culture with notochordal cells, conditioned medium and hypoxia
Source: BMC Musculoskelet Disord. 2014 Dec 11;15:422. doi: 10.1186/1471-2474-15-422 (PMC4295479; doi:10.1186/1471-2474-15-422)

gene expression bovine NPC

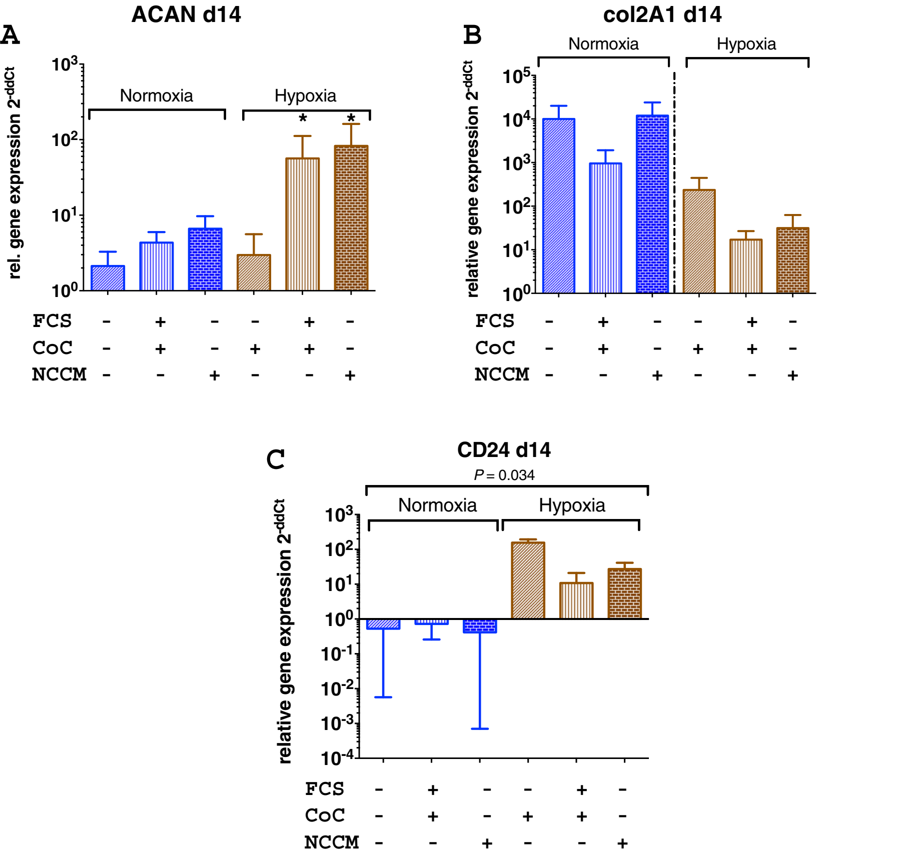

Supplement: Supplementary file 8 — Authors’ original file for figure 7 [file 12891_2014_2382_MOESM8_ESM.pdf]
